# Uncovering today’s rationalistic attunement

**DOI:** 10.1007/s11097-021-09728-z

**Published:** 2021-02-10

**Authors:** Paul Schuetze, Imke von Maur

**Affiliations:** Universitiy of Osnabrueck, Institute of Cognitive Science, Osnabrueck, Germany

**Keywords:** Rationalization, Attunement, Affect, Cultural affect, Situatedness, Episteme, Affectivity

## Abstract

In this paper, we explore a *rationalistic orientation* in Western society*.* We suggest that this orientation is one of the predominant ways in which Western society tends to frame, understand and deal with a majority of problems and questions – namely in terms of mathematical analysis, calculation and quantification, relying on logic, numbers, and statistics. Our main goal in this paper is to uncover the affective structure of this rationalistic orientation. In doing so, we illustrate how this orientation structures the way not only individuals but society as a whole frames and solves problems. We firstly point towards some exemplary instances of the rationalistic orientation*,* specifically regarding *science*, *society,* and *lifeworld practice*. Crucially, we argue that the rationalistic orientation is not merely based on a set of beliefs we could easily correct; but rather, that it is an *affective* condition tacitly shaping our engagement with the world in an encompassing way. Relating to the work of Martin Heidegger, we argue that what we have called an orientation in the beginning is in fact a *rationalistic attunement*. This attunement fundamentally shapes the pre-reflective level of how individuals approach the world. We elaborate this claim by showing how the rationalistic attunement concretely manifests in tangible socio-material affect dynamics. In the end, we motivate a critical stance towards this attunement, providing the ability to reflect upon and question instances where this way of framing and solving problems is counterproductive.

## Introduction

Should we vaccinate our children? What is the best care for a woman during pregnancy and birth? What are the necessary steps to reduce CO2 emissions? How do we achieve the best education in schools and universities? These are only a few examples of publicly debated questions where it is common practice to refer to science to get an adequate answer (Meyer and Jepperson 2000).^1^ Science has become a valuable guideline regarding many areas of our daily lives – be it concerning the questions above, the self-tracking fitness watch relying on scientific data, the apps many use to find the best sleep, or the numerous How-to manuals explicitly advertised with the predicate “scientifically proven”. The reason for this appears to be a public trust in scientific methods and practices. This trust in the scientific approach is not surprising if we look at how scientific research initiates progress and provides valuable knowledge. In times of climate change and pandemics, such as the COVID-19 outbreak, it is more than important to refer to scientific results and to reinforce these findings. Particularly in the case of medicine, scientific progress not only improves the quality of, but often even saves our lives. It is needless to say that science facilitates a positive development of our society and the world at large. In so far, the above-described trust in science and the application of its results is justified.

Though, in this paper, we aim to show that the described public trust in science also is indicative of a fundamental way according to which humans address the world more generally. This way is more adequately phrased as a trust in what we denote a *rationalistic orientation* – namely to frame, understand, and deal with the world in terms of mathematical analysis, calculation, and quantification, often by relying on logic, numbers, and statistics. When we ascertain such a general orientation in Western society, the reader may have in mind prominent countertrends to such an orientation, trends such as homeopathy, alternative birthing, climate skepticism, and even conspiracy theories. Hence, it is important to realize that these trends do not contradict, but rather share the diagnosis of a rationalistic orientation. This becomes evident in their explicit turning away from said orientation. On the one hand, this may simply be the belief that other approaches, which are neglected by the rationalistic orientation, should also be taken into account when approaching certain problems. For instance, alternative birthing methods, outside the realm of purely rationalistic methods, provide a more holistic picture of childbirth. On the other hand, turning away from the rationalistic orientation can also result in the complete denial of all of its methods. There are, for instance, climate sceptics which simply reject the evidence for anthropogenic climate change; similarly, conspiracy theorists find truth in outlandish interpretations of the COVID-19 pandemic. However, despite the grave differences between these countertrends which must not be neglected, they have one thing in common: They are countermovements against the prevailing rationalistic orientation, which we, as a society, adopt to a large number of problems and questions.

To be clear, we are not suggesting that all of the countertrends mentioned above are warranted or express valuable criticism. Rather, we take these trends to be indicators of an initial problem. While the rationalistic orientation is undoubtedly helpful in many areas, e.g. scientific research, it is also problematic when excessively and inappropriately extended. It is exactly this problem which we can observe in today’s Western society. We argue that the rationalistic orientation brings with it serious problems, not least of which are reflected in the emergence of some of the countertrends seen above. There are also much more mundane examples of the reductive understanding that comes with the rationalistic orientation.

Suppose higher education as an example: The overall aim of comparing the results of students leads to the fact that their outcome and performance is put into numbers, making them easily measurable and quantifiable. The complex and encompassing process of education, in the sense of *Bildung,* is reduced to what can be analyzed in this particular way. Anything not fitting the numbers is disregarded. In multiple-choice exams, only limited types of questions can be addressed; thus, it is often a very specific capability – learning things by heart – which is evaluated. This determines the form and content of not only exams but also of lectures and seminars. In this example, we see that an overly rationalistic approach does not always provide suitable solutions in regard to every problem. But, it might even lead to poor results that fail to adequately address the relevant issues.

In this paper, we argue that to understand the extent to which a rationalistic orientation overly influences not only us as individuals but society as a whole, we need to uncover its underlying *affective* structure. In pursuing this goal, we first point towards some exemplary instances of what we call a rationalistic orientation*,* specifically in regard to *science*, *society* and *lifeworld practice*. We do so, based on the work of Terry Winograd and Fernando Flores (1986), as well as Max Weber, Theodor W. Adorno, and Max Horkheimer. We assemble these influential insights and reveal that they share an underlying pattern, which consists of particular assumptions guiding the rationalistic orientation. Crucially, we show that the rationalistic orientation so characterized is not merely based on a (set of) belief(s) we could easily correct; but rather, it is an *affective condition* tacitly shaping our relationships with the world. We illustrate this affective nature of the rationalistic orientation with reference to Martin Heidegger’s work on *attunements*, as well as recent work on the understanding of *relational affect*.

## The rationalistic orientation

### The rationalistic tradition in science

To illustrate the idea of a rationalistic orientation in today’s Western societies, in the following we focus on a few “flags” of this orientation. It is important to note that we do not aim at presenting a comprehensive picture of what the world is like. However, we believe that the examples we are presenting are not mere singularities, but that there are underlying commonalities. While we do not deny that there are many different orientations in Western societies, we specifically focus on one we deem to be especially prevalent: the rationalistic orientation and its implications.

We start with an example from the sciences. According to Terry Winograd and Fernando Flores, there is a so-called *rationalistic tradition* which “has been the mainspring of Western science” for quite some time (Winograd and Flores 1986, p. 14). Winograd & Flores argue that there is a distinct manner of framing the questions addressed by science – a specific way of pre-understanding, namely what they call the rationalistic tradition.^2^ Essentially, this allows only for a limited perspective on the objects of investigation. In practice, this means that scientific research, as it is done in the rationalistic tradition, usually “consists of setting up situations in which observable activity will be determined in a clear way by a small number of variables that can be systematically manipulated” (1986, p. 16). The typical scientific approach to a research question in the spirit of the rationalistic tradition is: finding identifiable objects and finding interaction rules which, in combination, provide an answer to the initial question (1986, p. 15).

This characteristic procedure can nicely be illustrated by looking at the field of cognitive science. In this area, it has been and still is commonplace to understand and model cognition as well as cognitive systems in terms of representational variables – i.e. finding identifiable objects – and the systematic manipulation of these variables – i.e. finding interaction rules (1986, pp. 23–26). This is apparent in the paradigmatic and popular examples of *the Blue Brain Project* and *the Human Brain Project*. The aim of the Blue Brain Project is to build a detailed reconstruction and simulation of the mouse brain, and eventually extend this to construct a human brain in terms of computer models based on supercomputer simulations (Blue Brain Project, n.d.-a). Essentially, the project’s goal is to “build digital reconstructions (computer models) of the brain” (Blue Brain Project, n.d.-b), i.e. to find an appropriate mathematical model that can then be implemented as a computational model (Blue Brain Project, n.d.-c). Similarly, the Human Brain Project wants to understand the brain and cognition in terms of “models and conceptual frameworks which can be tested and refined” with a major focus on abstract modeling, simulating, and computationally implementing the brain (Human Brain Project: Understanding Cognition n.d.).^3^

In line with these examples, it is evident that “the rationalistic tradition is distinguished by its narrow focus on certain aspects of rationality” (Winograd and Flores 1986, p. 8). Moreover, this “often lead[s] to attitudes and activities that are not rational when viewed in a broader perspective” (1986, p. 8). When we use the term “rationalistic” in this paper we thus argue that there is an overweighting of such so-called rational faculties, methods, or strategies like analyzing, using logics, or formalizing processes. We do not want to diminish science or argue against the importance of scientific results (especially in times of climate change sceptics and mainstream conspiracy theories), but we want to highlight how the above described rationalistic tradition in the sciences tacitly became an underlying orientation in Western society. In the words of Winograd & Flores, the rationalistic tradition is “a way of understanding”, more specifically, a “*pre*-understanding” (1986, p. 7) which determines the room of possibilities for how questions are raised, and accordingly which kinds of answers can be given.^4^ In the following, we point towards instances of the rationalistic orientation in societal developments, as well as in private lifeworld practices.

### The rationalistic orientation in society

What Winograd and Flores identify as a rationalistic tradition in science they also find fundamentally manifested in the way humans approach the world. In their book they indicate that the rationalistic tradition is not only restricted to the “hard sciences”, but it may also be found in other areas of our lives, e.g. in societal developments and lifeworld practices (Winograd and Flores 1986, pp. 14–37). To call attention to the former, we refer to the social and cultural sciences, particularly to the work of Max Weber. Prominently, at the beginning of the twentieth century Weber described the universal *rationalization of society –* a phenomenon he diagnosed in all areas of society, such as economy, politics, culture, art, even sexuality (Kaesler 2014). He describes this rationalization as an expansion of instrumental rationality and a process of systematization, a process of organizing the world, fueled by the growth and progress of science, technology, capitalism, and bureaucracy (Kaesler 2014; Taylor 1995; Weber 1919/ 1930, p. 16). More specifically, Weber highlights that structures formerly provided by religion and metaphysical worldviews have now been replaced by instrumental rationality, rational technological means, and calculative processes (Habermas 1981, p. 481; Taylor 1995). In its excessiveness, this led to the modern faith that everything in the world will be knowable someday (Ritzer 2012) – that everything can essentially be mastered by rational calculation (Weber 1919/ 1930, p. 17).^5^ Weber even goes on to call this the *disenchantment of the world* – the sweeping away of old meaningful structures (Taylor 1995). Thereby, he captures a process driven by science and technology by which one exterminates all mysterious and unpredictable parts of the world (Weber 1919/ 1930, p. 17; see also Kahlberg 1980, p. 1154).

According to Weber, a central and exemplary element of the rationalization of society is the increasing influence of *bureaucracy* — he “saw bureaucracy as the epitome” of rationalization (Ritzer 2012, p. 43; Weber 1946). In line with the calculative and quantitative rationalistic orientation, bureaucracy aims at breaking the world up into smaller, quantifiable individual parts, thus making them easier to control and to manage (Ritzer 2012, p. 44). This approach “strongly furthers the development of ‘rational matter-of-factness’” by which the world progressively becomes more controllable and organized (Weber 1946, p. 240). Bureaucracy, as an example of rationalization, governs modern society – the authority of people was replaced by bureaucracy, by the authority of nobody (Arendt 1958/ 1994, p. 45). By subjecting everything to the measures of instrumental rationality, calculability, and controllability, society situates itself in a supposedly controllable world. Yet, submitting to these measures compels people to be mere bystanders to a world which unfolds in front of them – they are deprived of their authority, they are subject to a “steel-hard casing” governed by instrumental rationality (e.g., Kaesler 2012; Taylor 1995). This goes to show, how a rationalistic orientation can be observed in overarching societal developments, and it points out the negative consequences this orientation brings along.

Supporting Weber’s analysis*,* Theodor W. Adorno and Max Horkheimer also analyze the rationalization of society in their *Dialectic of Enlightenment* (1944). Essentially, they provide their own “version of a Weberian account of the disenchantment of the world” (Bernstein 1999, p. 308). For instance, they elaborate how the dominant influences of instrumental rationality force the environment to become nothing but a means to an end – nature loses its value due to the disenchantment of the world (Taylor 1995). As such, Adorno and Horkheimer specifically highlight the connection between the processes of rationalization and the “domination of nature” (Honneth 1986, p. 53). With Weber providing the idea of a universal rationalization of society, Adorno and Horkheimer now specifically highlight the modern exploitation of nature due to the rationalistic capitalist, scientific and technical progress (e.g., Honneth 1986, pp. 53–54; Rutherford 2000, p. 18). Concluding the above, we observe a rationalization of society in many of its areas (Weber) which has long been diagnosed, for example, in the modern ways we interact with nature (Adorno & Horkheimer).

Of course, there are vastly different factors shaping society and the way it has developed. Yet, we argue that the above-described flags of a rationalistic orientation are not just distinct singularities, but they depict a societal trend. Even though these flags do not provide an encompassing view of society, it should nevertheless be recognized that they are interconnected (Meyer and Jepperson 2000, p. 104).

### The rationalistic orientation in lifeworld practice

The rationalistic orientation can also be observed in the more private and personal area of daily lifeworld practices. An example that picks up the developments shown above is the field of medicine as it effectively combines the areas of science and lifeworld practice. Take for instance the changes of practices in midwifery and childbirth (see also Lang 2019). To make childbirth as safe as possible is an important goal which led to an enormous decrease in infant mortality in the last centuries. But, the increasing influence of science, medicine, and technology did not only have a positive impact. As a consequence of these advances, the very active and self-determined experience of giving birth became an objectified medical intervention (e.g., Ovidie 2019). Childbirth nowadays tends to be presented as “an objective scientific process for medical study or a ‘condition’ of the pregnant woman” (Lupton 2013, p. 54). The possibility of access and control offered by “medico-scientific instruments” has led to a neglect of embodied experiences (Lupton 2013, p. 35; see also Duden 1998). Medical settings and procedures objectify the internal processes of women, which often leads them to feel alienated from their very own experience in pregnancy (Young 1984, p. 55). They feel to lose control over their own embodied sensations and “[t]hey thus have developed a third-person relationship to their wombs” (Lupton 2013, p. 61). For instance, feminist academic Alice Adams notes that “[i]t still disturbs me to realize that my reconception of myself as a mother was mediated at its deepest level by obstetric technology” (as quoted in Lupton 2013, p. 61). As such, the influence of instrumental rationality has resulted in the discontent of many pregnant women, who feel left alone and not appreciated as an experiencing, bodily subject, but rather as an unfeeling, disembodied object (Martin 1996).

Just as the examples above, the process of childbirth suffers from the reductionist rationalistic orientation. The experiences of the bodily subjects are neglected for the sake of control: the goal of a successful and safe birth has led to the compulsive need to measure every detail of the process. This rationalistic approach has caused severe feelings of insecurity among women during the birth of their child (see Duden 1998; Martin 1996; Metz-Becker and Anselm 2019; Ovidie 2019). Instead of trusting themselves and their bodily feelings, women explicitly demand certainty and security in external sources, such as scientifically approved facts or measurable data of their own bodies (e.g., Metz-Becker and Anselm 2019). Yet, this results in superficial and simulated security that is artificially fabricated by the rationalistic approach. A feeling of real, bodily security and an experience of comfort is denied by the deeply rooted trust and belief in rationalistic methods. This demonstrates that even though there are many benefits to a rationalistic orientation, there are also many negative effects that need to be recognized. An overly rationalistic orientation severely disregards important parts of human existence.

Taking up a more generic example of our lifeworld practices, in his *Minima Moralia*, Adorno observes a rationalistic orientation and its negative consequences in peoples’ everyday interactions. One of the aphorisms remarks that “we are forgetting how to give presents” (Adorno 1951/ 2005, p. 42). According to Adorno, this is due to an overthinking of the gift-giving process. People are excessively thinking about the purpose of the present, contemplating whether the present is of the right size and value, they are calculating and quantifying, carefully weighing each variable; due to this logical dissection, they are violating the entire process. This is because, as Adorno puts it, “real giving had its joy in imagining the joy of the receiver” (1951/ 2005, p. 42). Due to the overthinking that grows out of the rationalistic orientation, the process of gift-giving has become one of instrumental rationality and not one guided, for instance, by affective engagement. As a consequence, we are not properly appreciating the other person anymore, we are not “expending time, going out of [our] way, thinking of the other as a subject” (1951/ 2005, p. 42).

As we have demonstrated employing the flags highlighted above, there appears to be a rationalistic orientation present in science, society, and lifeworld practice. This may be summarized as a cultural tendency – as “the cultural accounting of society and its environments in terms of articulated, unified, integrated, universalized, and causally and logically structured schemes” (Meyer and Jepperson 2000, p. 102).

### Central underlying assumptions

On a closer look, there are certain aspects of the rationalistic orientation which can be captured by several underlying assumptions. Recognizing these tacit assumptions helps to explain how the above-mentioned examples are connected, and it reveals how they are grounded in an affective condition. At the core of these assumptions – and the heart of the rationalistic orientation – are, as we argue, one ontological and one epistemic premise. In the following, we will focus on these two assumptions because relevantly they seem to be at the basis of what we have outlined above.

When analyzing any phenomenon at all according to the rationalistic orientation, one of the first steps should be to identify the respective component objects and their rules of interaction. This atomistic strategy implies the *ontological* assumption that any phenomenon – ultimately the world at large – consists of interacting individual pieces. Hence, by committing to the rationalistic orientation one accepts, as we call it, an *atomistic assumption*: the world can be split into parts and then be analyzed in terms of these building blocks as well as the rules according to which they interact (Winograd and Flores 1986, p. 15). Appropriately enough, in science most research is conducted committing to precisely that premise. Relating to the example of cognitive science, the idea of trying to model and understand cognitive systems in terms of variables and rules is essentially based on this very assumption. Moreover, referring to Max Weber, one may also see this assumption at the heart of the long-grown bureaucracy, namely in the division of the world into smaller, more manageable building blocks. Even in the example of childbirth, this presupposition is manifested: birth is understood in terms of variables and numbers – the building blocks – which supposedly make the whole process measurable and controllable.

Directly related to this ontological atomistic assumption is the *epistemic* premise that we can always grasp the essence of these building blocks, as well as define the rules of interaction (Winograd and Flores 1986, p. 15). This means that when approaching a scientific problem, the general belief is not only that there are isolatable blocks that make up the given phenomenon. But, the conviction is that one can eventually reveal their existence, gain knowledge about their nature, and know their interaction rules. This *knowledge assumption*, as we call it, can again most prominently be seen in the sciences. Relating once more to the example of cognitive science, the common belief in this field is that if we only have the right tools we will be able to gain complete knowledge about how the brain and thus how the mind works. In that way, one can also observe the knowledge assumption at the core of what Weber called the disentchantment of the world. By trying to eliminate all mysterious matters in the world, the goal essentially is to know it all (Weber 1919/ 1930, p. 17). The same holds for the examples of childbirth and gift-giving, in both cases the intention is to dissect, quantify, and thereby control these very complex processes.

Both, the atomistic (ontological) and the knowledge (epistemic) assumption, can be found at the core of the different examples described in section two. Once more this highlights that these flags are interconnected. A rationalistic orientation can be identified in different domains of our lives (e.g., Meyer and Jepperson 2000; Ritzer 2012; Winograd and Flores 1986). Of course, it is not in the scope of this paper to give a detailed analysis of the culture and history of Western society. Thus, the above represents a selected portion of views and examples that aim at revealing that there is what we call a rationalistic orientation.

## The rationalistic attunement

### The affective nature of the rationalistic orientation

The two assumptions presented above do not only indicate how the different flags are instances of the rationalistic orientation, but they also provide insights into why this orientation should be conceived of as an affective phenomenon. The affective nature of this orientation already substantiates in the atomistic and the knowledge supposition. This is nicely captured in William James’ paper *The Sentiment of Rationality* (1879). In his article James observes that when we are trying to make sense of the world, “the transition from a state of puzzle […] to rational comprehension is full of lively relief and pleasure” (James 1879, p. 317). He obtains that humans have “the *need of unity* and the *need of clearness*” when they try to understand the world (James 1879, p. 325; emphasis added).^6^ Once clearness and unity are achieved, the person feels relief and is satisfied. “This feeling of sufficiency of the present moment […] is […] the Sentiment of Rationality” (James 1879, p. 317). And since we always make sense of the world in some way, as “[t]he facts of the world […] are always before us, […] they should be conceived in such a way as to satisfy the sentiment of rationality” (James 1879, p. 318).

In effect, this relates to the two assumptions introduced above, i.e. the atomistic assumption: the world can be divided into building blocks including their rules of interaction; and the knowledge assumption: the nature of these blocks and rules is knowable. The need for clearness is manifested in “the passion for distinguishing; it is the impulse to be acquainted with the parts” (James 1879, p. 322). As such it is analogous to the atomistic assumption, the dissecting of the world into building blocks. The need of unity is characterized by the “pleasure at finding that a chaos of facts is at bottom the expression of a single underlying fact” (James 1879, p. 320). Therefore, it resembles the knowledge assumption, the faith that “we can know it all”. Additionally, the passion for unity emphasizes the longing for simplification within the explanatory structure of the blocks and their interaction rules.

The idea of a sentiment of rationality fittingly demonstrates the affective nature of the rationalistic orientation. The above-presented assumptions are not mere beliefs, but they are substantiated in an affective engagement with the world; according to James, these are feelings of relief, pleasure, and satisfaction. The rationalistic orientation is not manifested in several rules which humans simply follow, but it is embedded in how humans affectively engage with the world. What James calls the sentiment of rationality serves as the starting point for uncovering the affective nature of the rationalistic orientation. However, we do not share his view, but we use it as an outset for our explanations while diverging from it substantially. In our view, James puts too much focus on the feelings of relief, pleasure, and satisfaction. For him, the need for unity and the need for clearness are almost like character traits, which different people might possess and aim for. What is missing, is a proper understanding of how the cultural tendency of the rationalistic orientation concretely manifests in the subject. We argue that the rationalistic orientation is not a disposition which people possess in various ways (e.g. the need for unity or clearness) and which they strive to satisfy. Rather, we focus on how the rationalistic orientation is a cultural and affective phenomenon which implicitly influences all of us, and which we do not actively aspire to fulfill. Thus, while James’ account gives a perfect introduction to how the rationalistic orientation manifests in affective phenomena, we have a different goal: we understand the rationalistic orientation as an underlying cultural affective engagement with the world*.* Only when identifying it as such, the extent can be seen to which this affects not only individuals but society as a whole on a very fundamental level. By drawing on the work of Martin Heidegger, the next section illustrates how the rationalistic orientation can be understood not only as a (set of) belief(s) but as an affective way of approaching the world.

### Being attuned to the world

In the following, we make use of specific concepts Heidegger introduces and apply them in ways that are already abstracted from the original role they play in his work. We build on previous research by, for example, Matthew Ratcliffe, Hubert L. Dreyfus, and Jan Slaby who have applied and developed these concepts inspired by Heidegger. By transporting them into a new theoretical area, they provide the tools for a political discourse about the fundamental ways in which politics, public discourses, and social narratives are set up. Firstly, we illustrate Heidegger’s concept of *attunement* before going on to apply it to the rationalistic orientation.

One of the core insights of Heidegger’s philosophy is that we always experience the world through the lens of an affective sensibility [“*Befindlichkeit*”], so-called attunement (Heidegger 1927/ 1967, p. 137–140).^7^ According to Heidegger, the world necessarily discloses itself to us as affectively mattering in one way or another (Thonhauser 2019, p. 105). In other words, we always find ourselves situated in the world in terms of affective significance relationships, and this “primordial sense of significance is constituted by *Befindlichkeit*” (Thonhauser 2019, p. 105; see also Slaby 2017, pp. 11–14;). More specifically, attunement is the condition for different “moods” [“*Stimmungen*”]^8^ to present the world to us (Fuchs 2013); these moods *attune* [“*stimmen*”] us to the world in a certain way (Heidegger 1966, p. 24). Since different moods color the lens of attunement [“*Gestimmtheit der Befindlichkeit*”] (Heidegger 1927/ 1967, p. 137), in the following we will refer to these different colorings of attunement as different attunement*-s* [“*Befindlichkeit-en*”], e.g. the attunement of fearfulness [“*Befindlichkeit des Fürchtens*”] (1927/ 1967, p. 137).^9^ To get an idea of what attunements are, consider the following example from Adorno’s *Minima Moralia* (Adorno 1951/ 2005). The lines in one of his aphorisms perfectly capture how attunements*,* as a form of affective sensibilities, influence our experiences of the world:

We can tell if we are happy by the sound of the wind. It warns the unhappy man of the fragility of his house, hounding him from shallow sleep and violent dreams. To the happy man it is the song of his protectedness: its furious howling concedes that it has power over him no longer. (Adorno 1951/ 2005, p. 49).^10^

Although written in a different context, this passage nicely portrays how a mood attunes us to the world. In this case, being happy specifies a vastly different attunement than being unhappy. The world presents itself in two entirely different ways, i.e. experiencing the wind as comforting and calming, versus experiencing it as frightening and dangerous. In essence, this example reflects what Heidegger means by attunements: “Attunements are not simply modes of coloring our experience, but rather serve a fundamental disclosive function.” (Thonhauser 2019, p. 104).

Now, what precisely does it means to be attuned [“*ge-stimmt*”] to the world? First of all, it has to be noted that it is not only our experiences of the world that are based upon attunements, but all of our thinking grows out of them – moreover, our very being is essentially built upon attunements (Heidegger 1989, p. 21). Since we are always attuned to the world in a certain way, we cannot escape this condition – we are never not attuned to the world (Heidegger 1992, p. 102; see also Bollnow 1941/ 2009, p. 37). Our very existence is constituted by attunements in the sense that they “constitute the background that structures our world-relatedness” (Thonhauser 2019, p. 106). We find ourselves in the world and we relate to it in a way that is conditioned by attunements; they essentially disclose the world to us (Slaby 2017, p. 14). As such, attunements are the existential groundwork of our being-in-the-world, they are a primordial mode of our being (Heidegger 1992, p. 101; see also Dreyfus 1991; Thonhauser 2019).^11^ The important point is that attunements constitute our being-in-the-world (Ratcliffe 2002, p. 289). For example, if a person is in the attunement of fearfulness, she makes sense of the world in terms of very particular significance relationships. Her being is oriented in a specific direction, such that she might, for instance, expect fearsome events to happen incessantly (Heidegger 1927/ 1967, p. 137; Ratcliffe 2013, p. 163). In summary, an attunement can roughly be described as the fundamental constitutive part of being-in-the-world which discloses the world to us in a certain meaningful way.

### Fundamental attunements

How are attunements and the rationalistic orientation related? According to Heidegger, there are more fundamental kinds of attunements than those mentioned above – attunements which attune all of us from the very ground up (Heidegger 1992, p. 103; see also Dreyfus 1991, p. 170). Generally, he calls these elementary phenomena “*Grundstimmungen*” (Heidegger 1989), which we will refer to as the *fundamental attunement* in the following.^12^ Fundamental attunements underlie our being on a much deeper level than ordinary attunements. They do not just affect the experiences and thoughts of single individuals, but they shape the perspective of a whole culture – so to say, cultural attunements which constitute being-in-the-world in entire communities (Slaby and Thonhauser 2019, p. 278). Thus, one might say that a fundamental attunement alters the room of possible thoughts and experiences (“*Möglichkeitsraum*”) of an entire culture (see Slaby 2011); it defines the room in which being “can happen” (see Heidegger 1992, p. 103, 248).

A fundamental attunement is not necessarily intelligible or even knowable, yet it principally constitutes our being (Heidegger 1989, p. 20–23, 1992, p. 103). This kind of attunement does not change from day to day, week to week, or year to year, but it characterizes whole epochs in history (Heidegger 1966; see also Dreyfus 1991, p. 170; Slaby and Thonhauser 2019, p. 278). In that sense, a fundamental attunement must not be confused with other affective phenomena, such as an *emotional culture* or a *collective emotional orientation* (e.g.: Bar-Tal 2001; de Rivera 1992). These concepts are specifically targeted towards describing emotional tendencies in certain societies and cultures, whereas the idea of a fundamental attunement explains how essential aspects of our being, e.g. thinking, experiencing as well as emoting, are necessarily constituted by an underlying existential affective condition.

Here, it is of help to take into account a couple of parallel claims, namely what Michel Foucault introduces as *epistemes* (Foucault 1966/ 2005; Rouse 2005), and what Thomas Kuhn refers to as *paradigms* (Kuhn 1962; Bird 2015). These may be seen as somewhat analogous to the case of the fundamental attunement. In that sense, an episteme refers to a period in time during which our knowledge of the world is grounded in a specific set of assumptions (Foucault 1966/ 2005). Similarly, a paradigm indicates a certain collection of methods and prototype procedures which guide scientific research and the way knowledge is created (Kuhn 1962, p. 8). Interestingly, at this point, it should be mentioned that Heidegger also outlines such historical epochs in a different context of his work, namely in his account of the history of metaphysics (Dill 2017). In that regard, similar to knowledge in Foucault’s work and science in Kuhn’s writing, Heidegger describes the development of metaphysics in Western society and how it is transformed during different epochs in history (2017, pp. 295–296).^13^

For the aim of this paper, the important point is neither the content of these concepts, nor the specificity of the epochs they depict, but the very fact that Foucault, Kuhn, and Heidegger observe certain episodes in time which are contingently based upon particular assumptions, methods, and even metaphysics. Because assumptions, methods, and metaphysics may change over time, a particular epoch can always be characterized by its underlying suppositions and practices. If these mechanisms change, one may talk of a *paradigm shift* resulting in a new episteme (Foucault 1966/2005; Kuhn 1962). Relating this to the concept of fundamental attunements, we may already see the apparent analogy. Certain underlying assumptions and methods lead to a particular way of creating knowledge and thus lead to a specific kind of knowledge. In the same way does the fundamental attunement, during a particular period in time, impact the way we are in the world, i.e. the space of available concrete ways of thinking about and experiencing of the world.

In that sense, an episteme’s assumptions, a paradigm’s methods, and a fundamental attunement all have an analogous impact on a culture during a certain epoch. All these concepts frame the same issue, yet, they imply a different focus. While epistemes and paradigms emphasize the influence of knowledge, discursive structures, beliefs, and conceptions, fundamental attunements shine light on the affective grounding of human existence during certain epochs. Hence, the concept of attunements, as distinguished from paradigms and epistemes, has its asset in explicitly addressing the existential affective conditions underlying these epochs.

According to Heidegger, one can identify different underlying fundamental attunements during various periods in history (Heidegger 1966). These are cultural sensibilities which shape the way of how we are in the world during a specific epoch in time. Taking these explanations seriously, we can see how being-in-the-world during particular epochs in history is constituted by latent fundamental attunements (Heidegger 1966, p. 24, 1992, p. 104). Similar to a paradigm shift, if the fundamental attunements of an epoch change, new ways of being-in-the-world become feasible.

### The rationalistic attunement

In this section, we return to the rationalistic orientation and argue that this orientation frames a contemporary fundamental attunement which we will call the rationalistic attunement. How should the rationalistic attunement be understood? As we have explained before, Heidegger talks about different fundamental attunements during distinct epochs of history. For example, according to him, there was a different attunement during the time of the ancient Greeks than there is today (Heidegger 1966, 1989; see also Dreyfus 1991, p. 170). One dominant fundamental attunement “at the Greek beginning of philosophy was wonder (*Erstaunen*)” (Dreyfus 1991, p. 170) – the fundamental attunement “of beginning, birth, or the amazement that there is something rather than nothing” (Staehler 2007, p. 420). The modern fundamental attunement is supposedly one of alarm or doubt (Dreyfus 1991, p. 170; Heidegger 1966, p. 27). However, at this point, we deviate from Heidegger because we do not share his belief on such concrete and singular fundamental attunements during certain periods in time. In our view, there are always different fundamental attunements at play, which may vary in complexity. We keep as a starting point that one recent fundamental attunement was coined around the time of Descartes (Heidegger 1966, p. 27). In the following, we, therefore, retrace the central assumptions of the rationalistic orientation to Descartes, and so, we make visible the historicity of this orientation. To understand the connection between Descartes and the rationalistic orientation, we want to bring to mind a part of his second Meditation:

I shall proceed by setting aside all that in which the least doubt could be supposed […] and I shall ever follow in this road until I have met with something which is certain. (Descartes 1641/ 1911, p. 9)

It is this cartesian attitude of doubting anything at all which leads Heidegger to hold that one of the modern fundamental attunements has its starting point in Descartes’ Meditations. While this particular attitude is one aspect of what Descartes describes in his Meditations, there is another essential aspect; namely, that from an attitude of doubt follows a *longing for certainty*. All-encompassing doubt leads to the goal of gaining certainty – finding something that cannot be contested, an ultimate foundation. It is this part which shows the historicity of the rationalistic orientation, as it brings to light its rootedness in Descartes’ doubt.

Yet, the longing for certainty is not restricted to Descartes, but it can widely be found in the ideals of the scientific revolution (Kittsteiner 2006, pp. 34–54); more specifically in the striving for certitude and truth which was central to the natural sciences at that time (Kittsteiner 1982). Following Galileo Galilei, the natural sciences adopted mathematics as their language to be able to describe and quantify the world with utmost certainty (Goff 2019, pp. 3–25). This suggests that the desire for certitude can broadly be found in various historical developments, most prominently in the growth of science originating around the time of the scientific revolution (see Goff 2019, pp. 3–25). Of course, this is not to say that there is a generally valid and linear development of history, which is guided by the longing for certainty and characterized by the growth of science. Naturally, the course of history is much more complex. The above should rather highlight that the longing for certainty originating around the time of Descartes and Galileo can be found in different historical developments. These instances are not coincidental, but connected and exemplify a cultural tendency.

This takes us back to the rationalistic orientation. The search for certainty is tightly linked to the atomistic and the knowledge assumption. Striving for certainty in the world means trying to comprehend every little part of it. In other words, to get rid of all doubt about the world, one needs to divide the world into blocks and rules which one can eventually know. One cannot leave any indeterminate part behind, everything needs to be atomistically dissected, partitioned, and knowingly analyzed. From this follows that the longing for certainty can also be found in the different examples we introduced in the beginning: It can be observed in the rationalistic tradition described by Winograd and Flores, in what Adorno and Horkheimer coined the domination of nature, in what Weber takes up as the faith that “we can know it all” (the disenchantment of the world), and it can even be observed in childbirth. In other words, the faith that everything can eventually be truly known is manifested in the rationalistic orientation and can be found in various areas of life, e.g. science, society, and lifeworld practice (e.g., Bird 2015, p. 1–2; Heidegger 1966, p. 27; Steiner 1991, p. 88; Winograd and Flores 1986, p. 30).

Crucially, this orientation is affective. And so, if we take James’ sentiment of rationality as the outset, we can now properly specify the affective nature of the rationalistic orientation with the help of Heidegger’s notion of fundamental attunements*.* The rationalistic orientation structures how people make sense of their surroundings, it discloses the world to them in a specific way, it is an underlying affective condition. This means that we can phrase the affective nature of the rationalistic orientation as “the attunement of trust in the always accessible absolute certainty of knowledge” (Heidegger 1966, p. 27).^14^ This is essentially a fundamental attunement of the rationalistic orientation, manifested, for instance, in the atomistic and the knowledge assumption: It is the rationalistic attunement*.*

### Connecting the cultural (macro) and the individual (micro) level

To make this last point explicit, we want to illustrate the particularities of how a fundamental attunement manifests in a whole culture, as well as in the subject. How do individuals habituate epochal patterns? Specifically, which mechanisms bring a fundamental attunement to the affective lives of individuals? To clarify these issues, we bring in the notion of relational affect. This concept specifies how a subject and its socio-material surroundings are connected and how this connection is influenced by cultural patterns.

Relational “affect is seen as a dynamic relationality between organic and non-organic bodies, comprising an ontological layer of reality” (Szanto and Slaby 2020, p. 2). Affect thus understood, enables a focus on the subtle dynamics which constitute being-in-the-world. For instance, an attunement colors the affect relations of a subject in a particular way, e.g. the happy man relates to the sound of the wind in a completely different manner than the sad man. This relational idea of affect goes back to Baruch Spinoza’s complex metaphysical framework of substance monism (Mühlhoff 2018). Without subscribing to the whole conceptual landscape of Spinoza, the essential point here is to recognize affect as a relational phenomenon which is constitutive of the individual subject (Mühlhoff 2018; Seyfert 2012). This idea is nicely expressed in terms of *relational affect dynamics* which illustrate how a subject is situated in the world, i.e. in its social and physical surroundings (Mühlhoff 2018, p. 20). These dynamics form the base layer on which the individual subject itself is built upon (Åhäll and Gregory 2015, p. 5). They describe the material and ideational relations unfolding “between […] ‘bodies’ whose potentialities and tendencies are thereby continuously modulated in mutual interplay” (Slaby et al. 2019, p. 4). In connection to fundamental attunements, this means that individual affect relations are substantially influenced by the respective attunement, e.g. the affect relations of individuals in Western society are shaped by the rationalistic attunement.

While Heidegger’s concept of fundamental attunements makes possible the connection of the cultural (macro) with the individual (micro) level in general, the in-between level (meso) remains vague. What is the relationship between the macro level, i.e. the rationalistic attunement in Western culture, and the micro level, i.e. the rationalistic attunement in concrete individuals? As indicated above, the concept of relational affect allows us to approach these questions and shine light on the connection between the macro and micro level.

The flags from science, society, and lifeworld practice we outlined above are prime examples of connectors between the cultural and the individual level. For instance, childbirth and midwifery are subject to macro level cultural developments, e.g. the increasing influence of measurable and quantifiable scientific findings, and at the same time, they function at the micro level as they integrate individuals, and subject them to cultural developments, e.g. pregnant women feeling the need to quantify and measure their own bodies. As such, midwifery and childbirth are mediators working on the meso level, bringing cultural processes to the individual; they are *social institutions* which mediate between the macro and the micro level. We understand these instances as institutions in a broad sense, namely, as “systems of established and embedded social rules” (Hodgson 2006, p. 18) – as established “complex social forms that reproduce themselves” (Miller 2019). For instance, language, law, science, the family, giving presents, table manners, midwifery, hospitals, universities, and schools are all social institutions (see Hodgson 2006, p. 2; Miller 2019). In our understanding, institutions are social systems with solidified structures that are enduring over time and space (Giddens 1984, p. 24); this includes structures of rules, such as table manners, as well as concrete organizations, such as hospitals. Crucially, such institutional structures involve habituated rules of thought and behavior, which must not be understood as pre-formulated prescriptions that can be consciously deliberated, but they should be conceived of as transformative patterns guiding interactions and modes of being (Hodgson 2006, pp. 4–7; Giddens 1984, pp. 17–18). For example, childbirth is guided by exactly such established and enduring patterns, e.g. where to give birth, what available tests one should do, what to buy for the baby. In short, an institution is a social structure in which individuals habituate rules and acculturate modes of being peculiar to the institution.

This means that by “structuring, constraining, and enabling individual behaviors, institutions have the power to mold the capacities […] of agents in fundamental ways” (Hodgson 2006, p. 7). Importantly however, these capacities are not only restricted to thoughts or behavior but also include affect relations – institutions shape the affective lives of agents.^15^ For instance, there are unique affect dynamics at play during childbirth. Women affect and are affected in specific ways during their pregnancy. These affect dynamics are shaped by institutional patterns: they are habituated within a social structure, e.g. in the relationships, appointments, advisers, and health staff women are surrounded by during their pregnancy. Such established structures endure time and space; and in virtue of their intrinsic rules, they direct interactions, thoughts, and behavior within them. As such, individuals enter institutions which already “reside in the dispositions of other individuals but also depend on the structural interactions between them” (Hodgson 2006, p. 7). The institutional structure is already out there and acts upon the individual, but the individual also shapes the institution as individual behavior and affective comportment are necessary to enforce and uphold the norms and practices of institutions (see also von Maur 2018).

This is how a fundamental attunement, a cultural affective condition, finds its way to the individual. The rationalistic attunement influences the affect dynamics within society and its institutions, and so, it also shapes the affect relations of the individuals within these institutions. Similar to midwifery and childbirth, all the other flags mentioned above, e.g. gift-giving, cognitive science, education, and bureaucracy, are forms of social institutions in which macro developments manifest and thereby find their way to the individual. Of course, all of the dynamics within those institutions are multifaceted, and as such, the above can only provide a limited overview of the mechanisms at play. Moreover, individuals also have their own orientations which are not always in line with cultural and institutional rules or practices. Nevertheless, while individuals can diverge from such general trends and can even go against them, think of a midwife fighting against rationalistic practices, this does not deny an overarching trend. In fact, due to the nature of institutions, this trend is often forced upon individuals, even if they wanted to go against it. Again, think of the midwife wanting to change her practices, but laws, insurance policies, and the pressure from colleagues do not allow her to do so. This shows how institutional structures enforce cultural developments in a way which allows for diverging orientations, yet integrates and embeds individuals and enforces a prevailing mode of being upon them. There may very well be contradicting orientations between macro and micro level, but macro-level trends manifested in institutions at the meso level are powerful modulators of individual attitudes. This means that the rationalistic attunement substantiates within the structures of institutions, and while leaving space for diverging relations, it nonetheless subjects individuals to particular affect dynamics. In that way, an individual’s affect relations are structured, constrained, but also enabled by the rationalistic attunement via the respective institutions.^16^

The above explicitly shows how the rationalistic orientation shapes the everyday affect relations of subjects: the affective relations of individuals are fundamentally influenced by a rationalistic orientation. This is mediated by affect dynamics within social institutions which connect the cultural and the individual level. Through this mediation, the rationalistic orientation frames how people are in the world. Crucially, this should be understood as the rationalistic attunement, a fundamental attunement which “is the source, and atmosphere in which, thinking and action occur” (Freeman and Elpidorou 2015, p. 681).

## Conclusion: Critical outlook

In this paper, we have highlighted various flags exemplifying a rationalistic orientation. We have suggested that this is not just an orientation or cultural tendency, but rather, it is a fundamental attunement, namely the rationalistic attunement. Yet, the question remains what to make of this insight. Of course, the rationalistic orientation brings with it many positive effects (see Meyer and Jepperson 2000). But, a rationalistically attuned being-in-the-world results in the neglect of many relevant issues, and it brings with it a vast amount of negative consequences which must not be disregarded.

Above, we have described how our being is fundamentally connected to developments in society and institutions. Lastly, we want to emphasize the critical stance towards the rationalistic attunement which we already got a glimpse of in the descriptions before. In essence, we argue that an exclusively rationalistic attunement neglects important aspects of the world. If one follows Heidegger, this argument is rather straightforward, for his phenomenological approach is saying that “by reducing the horizons of our world and our experience to mathematical objectifications, the essential character of both world and experience are betrayed” (May 2005, p. 288). By relying on measurements and by modeling the world in terms of variables in a working system, one loses the way the world really is. Particularly, one does not need to analyze underlying variables and functions to reach an understanding of humans. Instead, one has to realize that human existence consists of nothing but being-in-the-world (e.g., Dreyfus and Rabinow 1982). “It is not what lies beneath human existence that reveals what humans are; it is what they actually live” (May 2005, p. 290).

But, recalling the different flags presented above, we have seen numerous other reasons warranting a critical stance towards the rationalistic attunement governing Western being-in-the-world today. Based on the work of Winograd & Flores we have seen the restricted angle of view present in today’s sciences resulting from the rationalistic attunement. For instance, in modern cognitive science, a majority of research is focused on trying to mathematically and computationally model cognition and the human brain respectively. Other approaches are scarce and excluded from the mainstream interest. With a nod to Weber, Adorno & Horkheimer, we elaborated on the fundamental impacts of the rationalistic attunement on societal developments and how this deeply affects human existence. For instance, the rationale of a meaningful being and a greater sense in life is hard to locate in a disentchanted world, within an overarching calculative and rational structure. Adding on to that, with reference to the example of childbirth, we have shown how the rationalistic attunement substantially shapes being-in-the-world. A rationalistically attuned approach to pregnancy and childbirth results in pregnant women feeling left alone and disconnected from their bodies. They increasingly experience themselves as disembodied objects confronted with and subjected to a rationalistic apparatus.

In sum, all of the above examples clearly outline the negative effects of the rationalistic attunement. And as such, they conclude the critical stance of this paper. An excessively rationalistically attuned being-in-the-world neglects essential features of our existence, such as the subjective experience of pregnant women, or the joy of giving a gift; and it disregards alternative or new perspectives, such as diverse approaches to the study of cognition, or different ideas for societal and institutional development. For this reason, the current paper calls for a critical reflection on how we affectively engage with the world in today’s Western society.

## Data Availability

Not applicable.

## Footnotes

1 We are specifically talking about Western cultures and the proposed argument may therefore not necessarily transfer to other cultures.

2 To avoid misunderstandings: This term does not refer to the philosophical tradition of rationalism in the classical sense (as opposed to empiricism) and as being proposed by e.g. Spinoza or Descartes.

3 While there are alternative approaches, such as the Heideggerian AI (see e.g.: Dreyfus 2007), these ideas are scarce. The dominant strands of research are oriented towards classical Neuroinformatics and Artificial Intelligence.

4 Winograd’s & Flores’ “tradition” is similar to concepts like “episteme” (Foucault 1966/2005), “paradigm” (Kuhn 1962), “scientific communities” (Fleck 1935/1980) or “frames” (Goffman 1974; Butler 2009). We refer to epistemes and paradigms more detailed in Section 3.2.

5 A much more detailed analysis of Weber’s description of rationalization can for example be found in Kahlberg 1980.

6 James holds that clearness and unity always need to be in balance with each other (James 1879, p. 322). Some people overly strive for unity, others for clearness. We leave aside this tension since our aim is not to adopt James’ entire concept.

7 Different scholars use different translations. However, all of them relate to the same realm of concepts (see e.g.: Ratcliffe 2002; Dreyfus 1991). We will use the term “attunement” (following Matthew Ratcliffe) because we think it best covers the phenomenon we are concerned with.

8 What Heidegger means by *Stimmung* has a broader range than mood as contemporarily understood (Dreyfus 1991, p. 169; Stephan 2017). In that sense, *Stimmung* covers a spectrum of affective phenomena; for example, fear can be a *Stimmung* even though it is not a mood strictly speaking, but rather an emotion (Heidegger 1927/1967, pp. 134–142).

9 We use attunement-s because we want to stick with the picture of “being attuned” to the world. Therefore, we hold that different *Gestimmtheiten der Befindlichkeit* can be referred to as different attunements.

10 We are aware that Adorno has criticized Heidegger massively in his *Jargon der Eigentlichkeit*. However, this does not directly concern the current paper (see e.g.: Pocai 2006).

11 With the concept of “being-in-the-world”, we take up Heidegger’s terminology. By being-in-the-world we refer to a human being’s being-there, so to say – “Dasein *as* being-in-the-world” (Wheeler 2018). In that way, we want to emphasize the idea that a “separation of subject and object denies the more fundamental unity of being-in-the-world” (Winograd and Flores 1986, p. 31). Being-in-the-world refers to human existence as always already being situated in the world: “The interpreted and the interpreter do not exist independently: existence is interpretation, and interpretation is existence” (Winograd and Flores 1986, p. 31).

12 This might also be called “fundamental mood” or “ground-mood” (see Dreyfus 1991, p. 170; Ratcliffe 2002, p. 292). However, we refer to the different colorings of “*Befindlichkeit*” as ‘*attunements’,* i.e. different *“Gestimmtheiten der Befindlichkeit*. To stay true to this terminology, we stick to the term attunement throughout this paper.

13 Not all researchers necessarily share Heidegger’s interpretation of the history of metaphysics and science.

14 Translated from German: “*Die Stimmung der Zuversicht in die jederzeit erreichbare absolute Gewißheit der Erkenntnis*“(Heidegger 1956/1966, p. 27).

15 For a detailed description of *affect in institutions* as well as an assortment of relevant literature see Slaby 2020.

16 The mismatch between individual and cultural orientations of course bears the potential for conflict between macro, meso, and micro level. However, going into detail in that regard is beyond the scope of this paper.
